# Molecular Integrity of Mitochondria Alters by Potassium Chloride

**DOI:** 10.1155/2015/647408

**Published:** 2015-12-10

**Authors:** Suman Mishra, Rajnikant Mishra

**Affiliations:** Biochemistry and Molecular Biology Lab, Department of Zoology, Banaras Hindu University, Varanasi 221005, India

## Abstract

Potassium chloride (KCl) has been commonly used in homogenization buffer and procedures of protein extraction. It is known to facilitate release of membrane-associated molecules but the higher concentration of KCl may affect the integrity of mitochondria by breaching the electrostatic force between the lipids and proteins. Therefore, it has been intended to explore the effect of KCl on mitochondrial proteome. The mitochondria were isolated from the mice liver and sub-fractionated into mitochondrial matrix and outer mitochondrial membrane fraction. The fractions were analysed by denaturing polyacrylamide gel electrophoresis (PAGE) and 2D-PAGE. The analysis of ultrastructure and protein profiles by MALDI-MS and data-mining reveals KCl-associated alterations in the integrity of mitochondria and its proteome. The mitochondrial membrane, cristae, and the matrix proteins appear altered under the influence of KCl.

## 1. Introduction

Mitochondria, the power house of the cell, are double membrane-bound organelle. The membranes provide compartmentalization to biomolecules and critically regulate physiological functions of organelles and cells. Among macromolecules of membranes, both extrinsic (peripheral) and intrinsic (integral) proteins show more variability. Mostly, integral proteins serve as transporters or receptors and their extraction has been facilitated by ionic detergents [1]. Peripheral proteins remain loosely associated with membrane by ionic or hydrogen bonding and function as specific transporters to regulate homeostasis of metabolites. Conventionally, salts (monovalent and divalent) and nonionic or mild-detergents have been used in the homogenizing medium for extraction of proteins of plasma membrane and membranes of cell organelles but they may also induce swelling of lipid layer [2–4] and change in ionic permeability [5–7]. Since mitochondria are enriched with proteins regulating several critical metabolic processes like oxidative phosphorylation, redox reactions, fatty acid synthesis, *β*-oxidation, and citric acid cycle, the isolation procedure of mitochondria for basic or clinical studies would bear large impact on the activities of enzymes and proteins. Therefore, it has been intended to explore the effect of KCl on integrity of mitochondrial membrane. Results provide insight on KCl-associated influences on integrity of mitochondria and leakage of mitochondrial matrix protein.

## 2. Materials and Methods

### 2.1. Animals, Materials, and Isolation of Mitochondria and Mitochondrial Membrane-Bound Fraction

**Figure 1:**
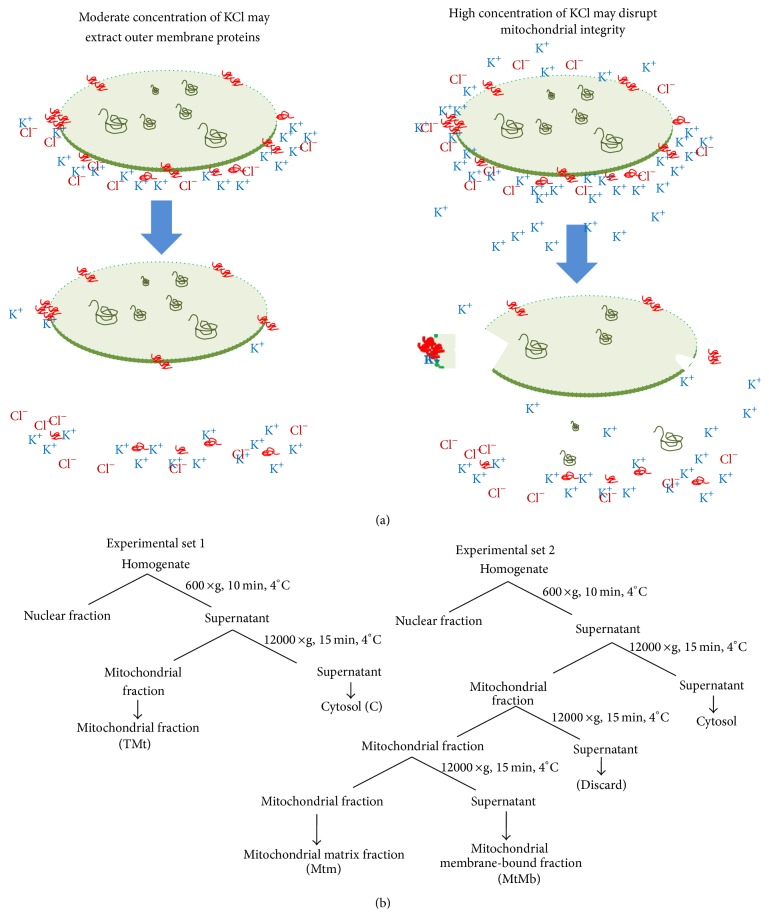
(a) demonstrates effect of KCl concentration on mitochondrial membrane. (b) Flowchart describes method of fractionation to study the effect of KCl concentration.

The adult mice (*Mus musculus*) of AKR strain were maintained with standard mice feed and drinking water at 25 ± 2°C in animal house facility of the department as per guidelines of the Institutional Animal Ethical Committee. Animals were sacrificed to obtain liver. All analytical grade chemicals were used. The 20% homogenate of liver was prepared in homogenizing buffer containing 30 mM Tris HCl (pH 7.2), 1 mM EDTA, 250 mM Sucrose, 50 mM Mannitol, and protease inhibitor cocktail (Sigma-Aldrich) using a Potter-Elvehjem type glass homogenizer with a motor-driven teflon pestle. Cytosolic and Mitochondrial fractions were isolated first without KCl in homogenizing medium and mitochondrial pellet was washed with homogenization buffer. Washed mitochondrial pellet was suspended in different concentrations of KCl (25 mM, 50 mM, 100 mM, 150 mM, and 200 mM) in homogenizing medium. Then, mitochondrial outer membrane-bound fraction (MtMb) and mitochondrial fractions (Mtm) were separated (Figure 1(b)). These fractions were used for protein profile analysis. Protein was estimated by the method of Bradford [8].

### 2.2. Protein Profile of Mitochondria and Mitochondrial Membrane-Bound Fractions

In order to analyse the influence of the concentration of KCl on the integrity of mitochondria and mitochondrial membrane-bound proteins, two sets of experiments were performed (Figure 1(b)). In first set (E1), different concentrations of KCl (0, 25 mM, 50 mM, 100 mM, 150 mM, and 200 mM) were used in homogenizing medium, and mitochondrial and cytosolic fractions were isolated, respectively. In second set (E2), mitochondria were isolated without KCl in homogenizing medium and mitochondrial pellet was washed twice with homogenization buffer to remove any cytosolic fraction. The mitochondrial pellet was then suspended in homogenizing medium containing 25 mM, 50 mM, 100 mM, 150 mM, and 200 mM KCl and mitochondrial membrane-bound fraction and mitochondrial fraction were separated, respectively. Proteins from different fractions were resolved on 15% denaturing polyacrylamide gel electrophoresis (SDS-PAGE) and proteins were visualized by CBB-R250 staining.

2D gel electrophoresis was performed to study the effect of KCl concentration on mitochondrial protein profile of the different fractions of the mitochondria. For 2D analysis the 100 mM KCl was selected because this concentration was found moderate and used in various extraction methods. The proteins of total mitochondria (before washing with 100 mM KCl), mitochondrial matrix (pellet of mitochondrial matrix after separating mitochondrial membrane), and mitochondrial membrane-bound fraction (supernatant after washing with 100 mM KCl) were precipitated with cold acetone and centrifuged at 3000 ×g for 5 min. The pellets were air-dried to remove the acetone. The proteins were solubilised in 0.125 mL of rehydration buffer (Bio-Rad 163-2106) and the sample was loaded on IPG strips 7 cm, pH 3–10 (Bio-Rad, 163-2000) gel in a side-down manner. The sample loaded strips were rehydrated for 12 hrs at 20°C. The rehydrated strips were focused in Protean i^12^ IEF cell (Bio-Rad cat number 1646002). The focused strips were equilibrated in equilibrating buffers I and II (Bio-Rad, 163-2107 and 163-2108) for 15 min each. The equilibrated strips were resolved in 10% denaturing-gel and proteins were visualized by silver staining. The gels were scanned and protein spots were analyzed by using PDQuest software version 7.1 (Bio-Rad) that include spot detection, quantification, background subtraction, and spot matching between multiple gels. Total spot intensity per gel was used to normalize spot intensities to compensate for variations between gel replicates. The relative spot volumes corresponding to total mitochondria (TMt), mitochondrial matrix (Mtm), and outer mitochondrial membrane-bound fraction (MtMb) were compared using Student's *t*-test. *p* values less than 0.05 were considered statistically significant. Likewise, the SDS-PAGE were analysed by Quantity One software (Bio-Rad) that includes band detection, quantification, background subtraction, and molecular weight prediction on the basis of standard molecular weight markers.

### 2.3. Analysis of Unique Protein Spot by MALDI/MSMS

The in-gel trypsin digestion of selected spot was performed before the MALDI analysis. The selected spot was excised and destained in washing solution containing 100 mM ammonium bicarbonate and acetonitrile (1 : 1 vol/vol). After destaining, spot was dehydrated in acetonitrile. The gel pieces were rehydrated and reduced in 10 mM DTT and incubated at 50°C for 30 min. After reduction, proteins spots were alkylated with 50 mM iodoacetamide in 100 mM ammonium bicarbonate buffer and incubated at room temperature for 30 min. Gel pieces were again washed and digested overnight at 37°C in trypsin solution (25 ng/*μ*L in 25 mM ammonium bicarbonate buffer). The peptides were extracted in extraction buffer (100% acetonitrile and 1% TFA in water, 1 : 1 vol/vol), suitably concentrated and analysed through MALDI MS/MS. Data generated were screened through MASCOT database (http://www.matrixscience.com/) and Panther software (http://www.pantherdb.org/).

### 2.4. Ultrastructural Evaluation of Mitochondria

The mitochondrial pellets of control (isolated without KCl) and KCl treated fractions (isolated with 100 mM KCl) were fixed in 2.5% glutaraldehyde and 1% paraformaldehyde in phosphate buffer (pH 7.5) at 4°C overnight. The fixed mitochondrial pellets were dehydrated through grades of alcohol (30% to absolute alcohol), and the preparation of block, sectioning, and staining for TEM analysis was executed at AIIMS, New Delhi. The images were further analysed by ImageQuant software (GE healthcare).

## 3. Results

### 3.1. The Optimal Level of Widely Used KCl Induces Leakage of Mitochondrial Proteins

**Figure 2:**
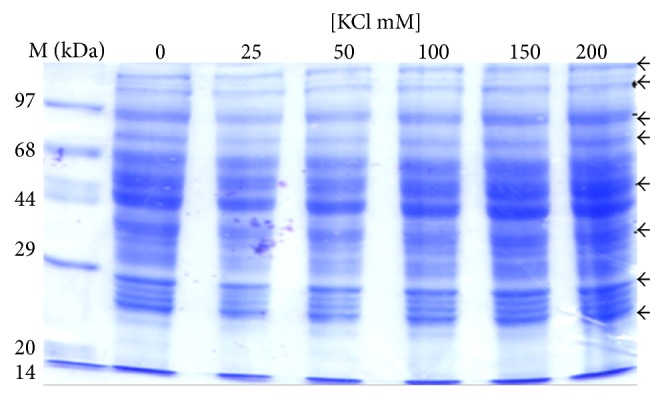
Effect of concentration of KCl on protein profile of cytosolic fraction (set 1). The protein profile of cytosolic fraction indicates the release of the membrane proteins showing increased intensity of bands near to 110 kDa, 97 kDa, 70 kDa, 68 kDa, 44 kDa, 38 kDa, 28 kDa, 25 kDa, and 23 kDa. The increase in intensity of these bands was probably due to protein released from the membrane of mitochondria and other subcellular organelles.

**Figure 3:**
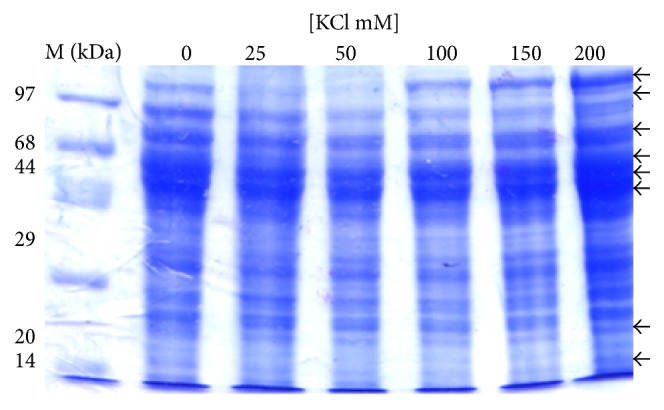
Effect of concentration of KCl on protein profile of mitochondrial fraction (set 1). The mitochondrial fraction shows decrease in intensity of polypeptides 110 kDa, 97 kDa, 70 kDa, 68 kDa, 66 kDa, 44 kDa, 20 kDa, and 14 kDa with increasing KCl concentration.

**Figure 4:**
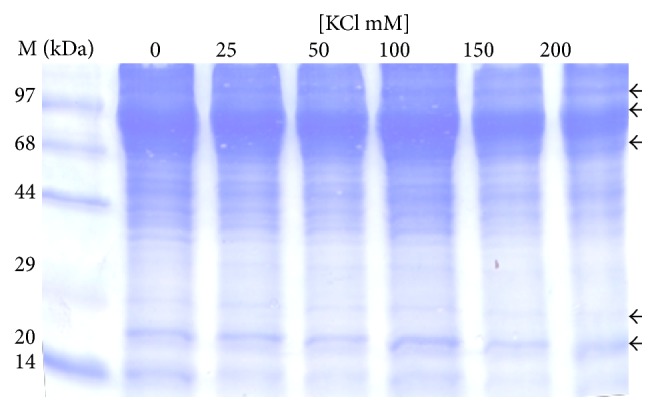
Effect of concentration of KCl on protein profile of mitochondrial matrix fraction (set 2). The protein profile of mitochondrial fraction shows decrease in intensity of some bands of 110 kDa, 96 kDa, 70 kDa, 23 kDa, and 20 kDa.

**Figure 5:**
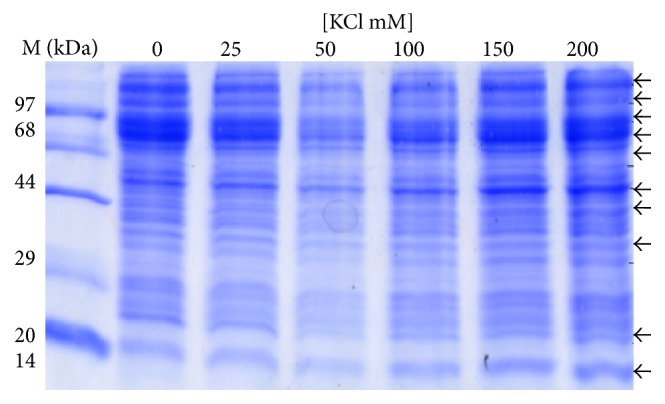
Effect of concentration of KCl on protein profile of membrane-bound fraction (set 2). The protein profile of the mitochondrial membrane-bound fraction shows polypeptides of 150 kDa, 120 kDa, 100 kDa, 97 kDa, 93 kDa, 88 kDa, 67 kDa, 50 kDa, 48 kDa, 44 kDa, 33 kDa, 23 kDa, and 20 kDa.

The analysis of protein profile of the cytosolic and the mitochondrial fractions showed increased intensities of bands of 110 kDa, 97 kDa, 70 kDa, 68 kDa, 44 kDa, 38 kDa, 28 kDa, 25 kDa, and 23 kDa in the cytosolic fraction (Figure 2) while lower levels of 110 kDa, 97 kDa, 70 kDa, 68 kDa, 66 kDa, 44 kDa, 20 kDa, and 14 kDa proteins were observed in the mitochondrial fraction (Figure 3). In second set of experiment (E2) progressive decrease in intensities of 110 kDa, 96 kDa, 70 kDa, 23 kDa, and 20 kDa was observed with increasing concentration of KCl (Figure 4). However, higher intensities of 150 kDa, 120 kDa, 100 kDa, 97 kDa, 93 kDa, 88 kDa, 67 kDa, 50 kDa, 48 kDa, 44 kDa, 33 kDa, 23 kDa, and 20 kDa bands in mitochondrial membrane-bound fraction was (Figure 5) evident with increasing KCl concentration.

**Figure 6:**
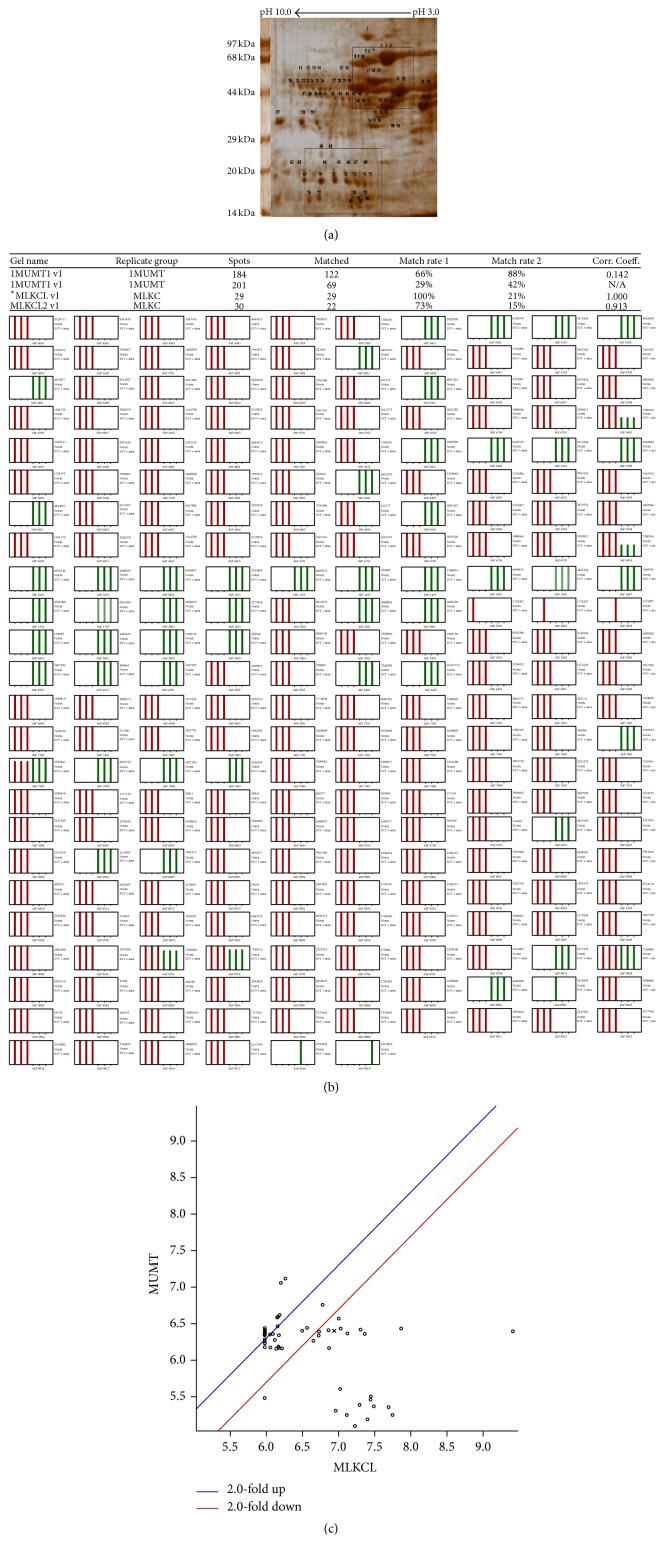
(a) Effect of concentration of KCl on 2D protein profile of total mitochondrial fraction. Selected area in rectangle (upper rectangle and lower rectangle) shows the variation of spots with the effect of KCl. (b) Spot intensity analysis of 2D protein profile of mitochondrial matrix fraction (MLW) in comparison to protein profile of mitochondrial membrane-bound fraction (MLKCl). (c) Correlation graph analysis of 2D protein profile of mitochondrial matrix fraction (MLW) in comparison to mitochondrial membrane bound fraction (MLKCl).

**Figure 7:**
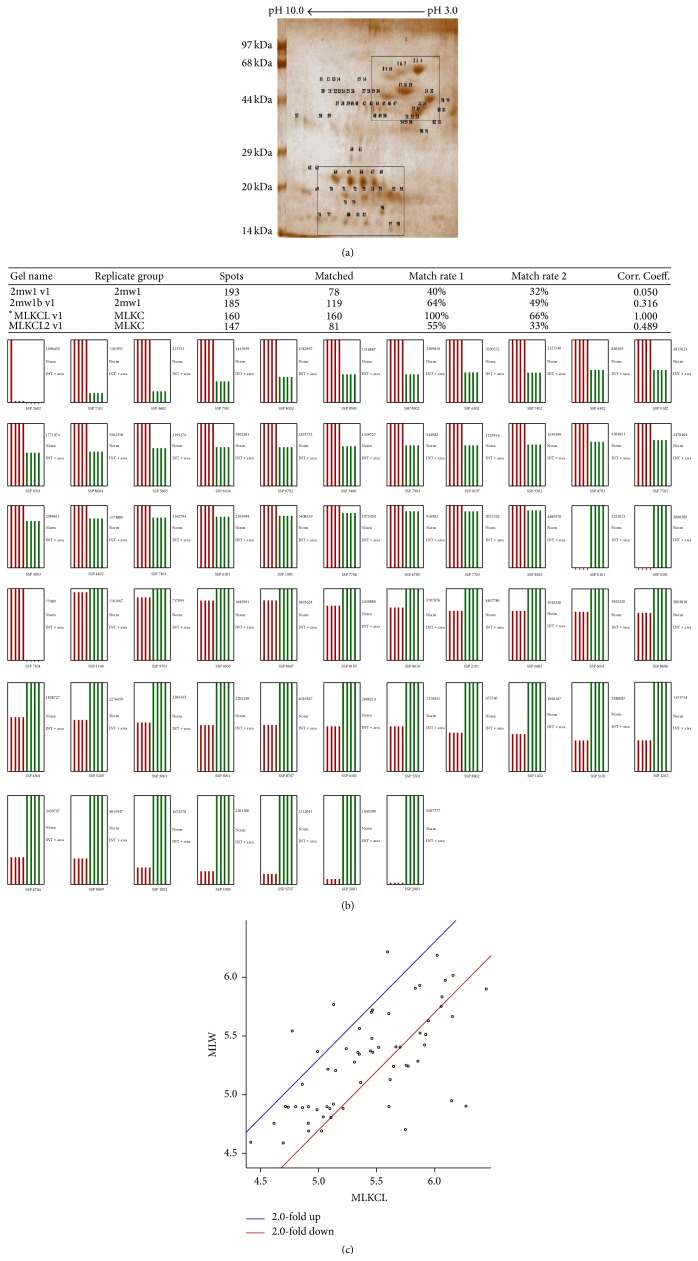
(a) Effect of concentration of KCl on 2D protein profile of mitochondrial membrane bound fraction. Selected area in rectangle (upper rectangle and lower rectangle) shows the variation of spots with the effect of KCl. (b) MS spectra of the unique spot of mitochondrial membrane-bound fraction. (c) Analysis of biological functions of proteins identified by MALDI/MSMS and their classification represented as pie chart.

**Figure 8:**
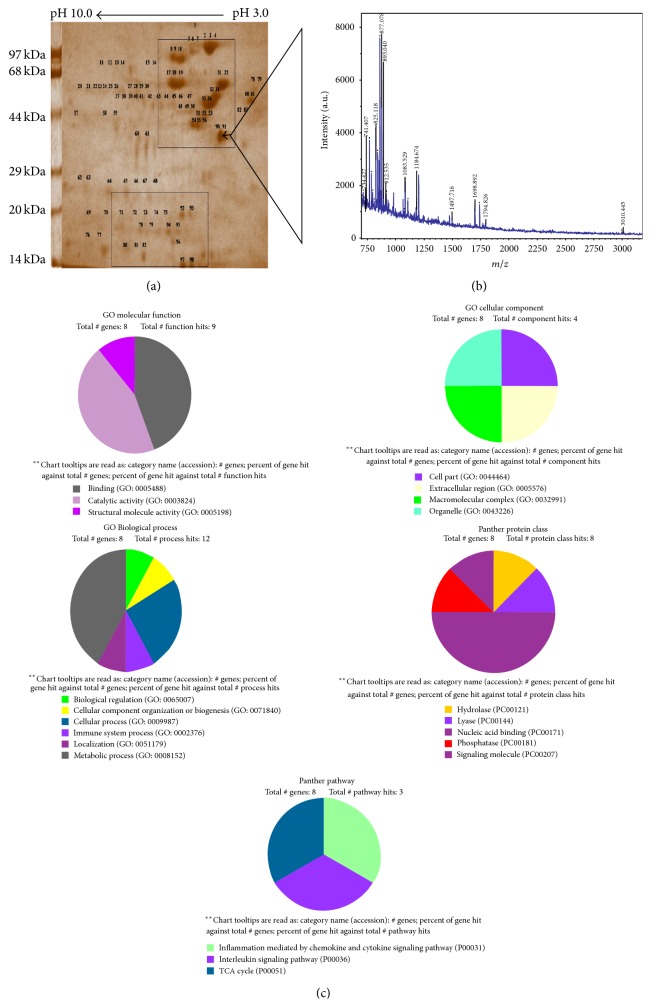
(a) Effect of concentration of KCl on 2D protein profile of mitochondrial membrane-bound fraction. Selected area in rectangle (upper rectangle and lower rectangle) shows the variation of spots with the effect of KCl. (b) MS spectra of the unique spot of mitochondrial membrane-bound fraction. (c) Analysis of biological functions of proteins identified by MALDI/MSMS and their classification represented as pie chart.

The analysis of 2D gel profile (Figures 6–8) revealed that the intensities of protein spots in matrix fraction were less than the membrane-bound fractions. Protein spots around pI3–6 and 44–97 kDa (upper rectangle) were significantly altered as compared to group of spots below 44 kDa and pI4–7 (lower rectangle). The number and intensities of spots were more in the membrane-bound fraction (Figure 8) than mitochondrial matrix fraction (Figure 7). Similarly, PDQuest analysis of the total mitochondrial matrix versus KCl solubilised fraction showed only 40% matching of the spots from the KCl solubilised fraction. However, the analysis of washed mitochondrial matrix versus KCl showed 60% matching with control gel. The intensities of several spots in the KCl solubilised fraction were higher by 2-fold in total mitochondrial and washed mitochondrial matrix fraction. The analysis of proteins represents translocase of outer mitochondrial membrane complex (TOM) and translocase of inner mitochondrial membrane complex (TIM), Bcl2, VDAC, Porins, and Acyl CoA Synthetase (76 kDa) and Hexokinase I (100 kDa); inner-membrane space proteins Cytochrome-C oxidase (14 kDa), inner-mitochondrial membrane proteins such as Succinate Dehydrogenase (70 kDa and 27 kDa), Aconitase (44 kDa), ATP Synthatase (56 kDa and 30 kDa), NADH Dehydrogenase (51 kDa, 30 kDa and 17 kDa), and Carnitine Acyl transferase I (88 kDa) represent association with mitochondrial membrane. However, Citrate Synthetase (44 kDa), Isocitrate Dehydrogenase (16 kDa), *α*-keto Dehydrogenase I (114 kDa), *α*-keto Dehydrogenase II (52 kDa), Fumarate Dehydrogenase (55 kDa), Malate Dehydrogenase II (33 kDa), Pyruvate Dehydrogenase (93 kDa), Fructose bisphosphate aldolase B (48 kDa), and PEP Carboxykinase (75 kDa) belong to mitochondrial matrix proteins (Figure 8).

### 3.2. Ultrastructural Changes in the Mitochondrial Integrity

**Figure 9:**
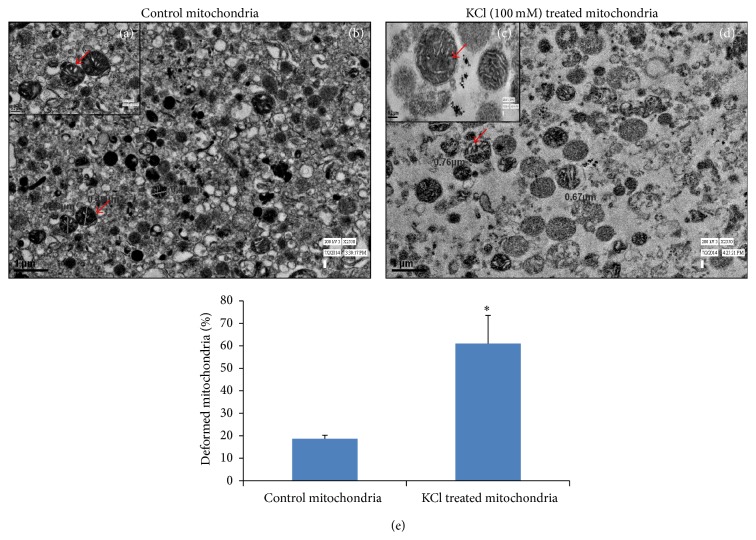
Transmission electron microscopic analysis of mitochondrial surface before and after the treatment of KCl showed mitochondrial swelling, altered morphology of cristae, and disruption in outer mitochondrial membrane after KCl treatment. (a) Ultrastructure of control mitochondria; (b) enlarged micrograph of isolated mitochondria; (c) ultrastructure of KCl treated mitochondria; (d) enlarged micrograph of KCl treated mitochondria; (e) histogram of % of deformed mitochondria of control and KCl treated mitochondrial fraction (percentage is calculated by dividing number of deformed mitochondria by total number of mitochondria). *∗* denoted the significance level *p* < 0.05 compared to control.

The ultrastructural analysis by TEM indicates KCl-dependent swelling of mitochondria and disruption of the outer mitochondrial membrane (Figures 9(a)–9(d)). The deformities in the cristae of KCl-treated mitochondria (Figures 9(c), 9(d), and 9(e)) were evident as compared to control.

## 4. Discussion

Observations provide insight into alterations in the integrity of mitochondria under routinely used concentration of potassium chloride (KCl). The analysis of unique spots of mitochondrial membrane-bound fraction and data-mining of proteins around pI3–6 and 44–97 kDa show significant alteration of proteins associated with enzymes and proteins of oxidative phosphorylation, pyruvate oxidation, and *β*-oxidation (Table 1). The observation indicates impact of KCl on the outer membrane-associated proteins or loosely associated proteins from 17 kDa to 50 kDa. These proteins appear to be Porins and transporters proteins. However, the enrichment of high molecular weight spots and medium to lower molecular weight spots indicates KCl-induced damage of mitochondrial integrity. The 2D analysis shows two fold increase in the intensity of membrane-bound fraction (Figure 8) in comparison to total mitochondria (Figure 6) and mitochondrial matrix (Figure 7). The optimal level of the KCl has been explained to breach the electrostatic bonds of the proteins to facilitate solubilization of proteins (Figure 1(a)) but the higher concentration of KCl may disrupt integrity of the membrane by modifying polarization of lipid bilayer, permeabilization of the mitochondrial membrane, and shrinkage of mitochondrial membrane (Figure 1(b)). Similarly salt-dependent modulation of Hexokinase-I and Cytochrome-C [9], integrity of the organelle membrane [2, 10–12], opening of mitochondrial permeability transition pore [6, 13, 14], and salt-induced pH homeostasis [7] have also been explained. The monovalent salts were also suggested to alter permeability of the membrane characteristics [4, 5, 10].

On data-mining, modulated proteins like translocases, Porins, and Acyl CoA Synthetase and Hexokinase, Cytochrome-C oxidase, Succinate Dehydrogenase, Aconitase, ATP Synthatase, and NADH Dehydrogenase represent association with mitochondrial membrane (Figure 8). However, Citrate Synthetase, Isocitrate Dehydrogenase, *α*-keto Dehydrogenases, Fumarate Dehydrogenase, Malate Dehydrogenase, Pyruvate Dehydrogenase, Fructose bisphosphate aldolase B, and PEP Carboxykinase belong to mitochondrial matrix. The alteration in the ultrastructure of mitochondria after the treatment of KCl supports mitochondrial swelling, altered morphology of cristae, and disruption in outer mitochondrial membrane as compared to control (Figure 9(a)). The KCl induced damage of mitochondrial outer membrane and extraction of outer mitochondrial membrane-associated proteins were also suggested by the disruption of electrostatic force between lipid and protein molecules. Similarly, tissue-specific mitochondrial proteome analysis was also identified [15]. Proteins like Fumarate hydratase and Fructose 2,6-bisphosphate aldolase B were modulated significantly by the KCl.

## 5. Conclusion

Potassium chloride, a monovalent salt and common component of different extraction procedures, affects mitochondrial integrity and proteomics of mitochondria. It affects both matrix and membrane-bound proteins.

## Figures and Tables

**Table 1:** List of identified proteins based on functional categories of PANTHER.

	Gene ID	Mapped IDs	Gene name Gene symbol ortholog	PANTHER family/subfamily	PANTHER protein class	Species
1	MOUSE|MGI=MGI=1923417|UniProtKB=Q9D384	21624627	U11/U12 small nuclear ribonucleoprotein 35 kDa protein; Snrnp35; Ortholog	U11/U12 SMALL NUCLEAR RIBONUCLEOPROTEIN 35 KDA PROTEIN (PTHR13952:SF6)	mRNA splicing factor	*Mus musculus*

2	MOUSE|MGI=MGI=95530|UniProtKB=P97807	226823367 13543801	Fumarate hydratase, mitochondrial; Fh; Ortholog	FUMARATE HYDRATASE, MITOCHONDRIAL (PTHR11444:SF1)	Lyase	*Mus musculus*

3	|MGI=MGI=96548|UniProtKB=P04351	7110653 387384 52658	Interleukin-2; Il2; ortholog	INTERLEUKIN-2 (PTHR11443:SF0)	Interleukin superfamily	*Mus musculus*

4	MGI=MGI=1859610|UniProtKB=Q56A08	26324390 38571790 117306436	G patch domain and KOW motifs-containing protein; Gpkow; Ortholog	G PATCH DOMAIN AND KOW MOTIFS-CONTAINING PROTEIN (PTHR15818:SF2)	Nucleic acid binding	*Mus musculus*

5	MOUSE|MGI=MGI=96832|UniProtKB=P19973	408357992	Lymphocyte-specific protein 1; Lsp1; Ortholog	LYMPHOCYTE-SPECIFIC PROTEIN 1 (PTHR18949:SF1)	—	*Mus musculus*

6	MOUSE|MGI=MGI=105381|UniProtKB=P14206	62024907 12846904 26344606 26350123	40S ribosomal protein SA; Rpsa; Ortholog	40S RIBOSOMAL PROTEIN SA (PTHR11489:SF10)	Ribosomal protein	*Mus musculus*

7	MOUSE|MGI=MGI=2181202|UniProtKB=A7UAK5	295293209	6-phosphofructo-2-kinase/fructose-2, 6-biphosphatase 3 splice variant 2; Pfkfb3; Ortholog	6-PHOSPHOFRUCTO-2-KINASE/FRUCTOSE-2,6-BISPHOSPHATASE 3 (PTHR10606:SF41)	Carbohydrate phosphatase, carbohydrate phosphatase	*Mus musculus*

8	MOUSE|MGI=MGI=1328362|UniProtKB=O88700	3370996	Bloom syndrome protein homolog; Blm; Ortholog	BLOOM SYNDROME PROTEIN (PTHR13710:SF70)	DNA helicase helicase	*Mus musculus*
